# Rapid Scale-up of an Antiretroviral Therapy Program Before and During the COVID-19 Pandemic — Nine States, Nigeria, March 31, 2019–September 30, 2020

**DOI:** 10.15585/mmwr.mm7012a3

**Published:** 2021-03-26

**Authors:** Emilio Dirlikov, Ibrahim Jahun, Solomon F. Odafe, Ogbanufe Obinna, Chibuzor Onyenuobi, Mgbakor Ifunanya, Timothy A. Efuntoye, Nguhemen Tingir, Uzoma Ene, Ayodele Fagbemi, Chidozie Meribe, Orji Bassey, Adeola Ayo, Omodele Johnson Fagbamigbe, Joy Amafah, Moyosola Bamidele, Matthias Alagi, Ademola Oladipo, Ibrahim Dalhatu, McPaul Okoye, Dennis Onotu, Jerry Gwamna, William A. Abrams, Deborah A. Conner, Anuli Nwaohiri, Deborah Carpenter, Ugonna C. Ijeoma, Sarita Shah, Laura I. Tison, Minesh Shah, Helen Chun, Michelle Williams-Sherlock, Andrew T. Boyd, Pamela Bachanas, Akudo Ikpeazu, Gambo Gumel Aliyu, Tedd Ellerbrock, Mahesh Swaminathan, CDC Nigeria ART Surge Team, CDC Nigeria ART Surge Team, Kathleen FitzGibbon, Mark Giambrone, Andrew Abutu, Ian Fellows, Lisa Murie, David Miller, Obinna Nnadozie, Ray Shiraishi, Viva Thorsen, Opeyemi Adebayo, Patrick Dakum, Charles Mensah, Fadimatu Mishara, Olupitan Olayemi Kinmilola, Benjamin Pillatar, Chukwu-Emeka Okolo, Tarfa Verinumbe, Yakubu Sambo, Fidelis Yillong, Oluwaseun Abe, Olaitan Achor, Musa Bashir, Stephen Bature, Emily Madina, Bolanle Oyedelun, Fabian Bassey, Amana Effiong, Alimigbe Francis, Dorcas Magbadelo, Charles Okolie, John Okpanachi Oko, Olanrewaju Olayiwola, Eugene Onu, Chukwudi Onwuchekwa, Mikhail Obaje, Prosper Okwonkwo, Thomas Usha, Evans Ejimkaraonye, Chiagozie Achebe, Sylvia Adebajo, Johnson Alonge, Moses Asiozi, Nelson Attah, Nnabundo Musei, Prosper Onyekachi, Kristen A. Stafford

**Affiliations:** ^1^Division of Global HIV & TB, Center for Global Health, CDC; ^2^Division of Global HIV &TB, Center for Global Health, CDC Nigeria; ^3^National AIDS and STIs Control Programme, Abuja, Nigeria; ^4^National Agency for the Control of AIDS, Abuja, Nigeria.; U.S. Department of State; U.S. Department of State; CDC; CDC; CDC; CDC; CDC; CDC; CDC; Institute of Human Virology, Nigeria; Institute of Human Virology, Nigeria; Institute of Human Virology, Nigeria; Institute of Human Virology, Nigeria; Institute of Human Virology, Nigeria; Institute of Human Virology, Nigeria; Institute of Human Virology, Nigeria; Institute of Human Virology, Nigeria; Institute of Human Virology, Nigeria; Institute of Human Virology, Nigeria; Centre for Integrated Health Programs, Nigeria; Centre for Integrated Health Programs, Nigeria; Centre for Integrated Health Programs, Nigeria; Centre for Integrated Health Programs, Nigeria; Centre for Integrated Health Programs, Nigeria; Centre for Integrated Health Programs, Nigeria; Catholic Caritas Foundation Nigeria; Catholic Caritas Foundation Nigeria; Catholic Caritas Foundation Nigeria; Catholic Caritas Foundation Nigeria; Catholic Caritas Foundation Nigeria; Catholic Caritas Foundation Nigeria; Catholic Caritas Foundation Nigeria; Catholic Caritas Foundation Nigeria; Catholic Caritas Foundation Nigeria; AIDS Prevention Initiative in Nigeria, Public Health Initiatives; AIDS Prevention Initiative in Nigeria, Public Health Initiatives; AIDS Prevention Initiative in Nigeria, Public Health Initiatives; AIDS Prevention Initiative in Nigeria, Public Health Initiatives; Center for International Health, Education, and Biosecurity and Maryland Global Initiatives Corporation, University of Maryland; Center for International Health, Education, and Biosecurity and Maryland Global Initiatives Corporation, University of Maryland; Center for International Health, Education, and Biosecurity and Maryland Global Initiatives Corporation, University of Maryland;; Center for International Health, Education, and Biosecurity and Maryland Global Initiatives Corporation, University of Maryland; Center for International Health, Education, and Biosecurity and Maryland Global Initiatives Corporation, University of Maryland; Center for International Health, Education, and Biosecurity and Maryland Global Initiatives Corporation, University of Maryland; Center for International Health, Education, and Biosecurity and Maryland Global Initiatives Corporation, University of Maryland; Center for International Health, Education, and Biosecurity, Institute of Human Virology, University of Maryland.

In 2018, an estimated 1.8 million persons living in Nigeria had HIV infection (1.3% of the total population), including 1.1 million (64%) who were receiving antiretroviral therapy (ART) (*1*). Effective ART reduces morbidity and mortality rates among persons with HIV infection and prevents HIV transmission once viral load is suppressed to undetectable levels (*2*,*3*). In April 2019, through the U.S. President’s Emergency Plan for AIDS Relief (PEPFAR),* CDC launched an 18-month ART Surge program in nine Nigerian states to rapidly increase the number of persons with HIV infection receiving ART. CDC analyzed programmatic data gathered during March 31, 2019–September 30, 2020, to describe the ART Surge program’s progress on case finding, ART initiation, patient retention, and ART Surge program growth. Overall, the weekly number of newly identified persons with HIV infection who initiated ART increased approximately eightfold, from 587 (week ending May 4, 2019) to 5,329 (week ending September 26, 2020). The ART Surge program resulted in 208,202 more HIV-infected persons receiving PEPFAR-supported ART despite the COVID-19 pandemic (97,387 more persons during March 31, 2019–March 31, 2020 and an additional 110,815 persons during April 2020–September 2020). Comprehensive, data-guided, locally adapted interventions and the use of incident command structures can help increase the number of persons with HIV infection who receive ART, reducing HIV-related morbidity and mortality as well as decreasing HIV transmission.

In April 2019, CDC launched an 18-month ART Surge program to rapidly increase the number of persons with HIV infection receiving ART.^†^ CDC and its four implementing partners^§^ established incident command structures to manage operations in nine Nigerian states (*4*) with a combined estimated ART coverage gap of 320,921 persons with HIV infection not on ART, according to the 2018 Nigeria HIV/AIDS Indicator and Impact Survey (NAIIS) (*1*) (Benue, 35,623 of 320,921 [11%]; Delta, 34,325 of 320,921 [11%]; Enugu, 29,623 of 320,921 [9%]; Federal Capital Territory, 1,169 of 320,921 [0.4%]; Gombe, 684 of 320,921 [0.2%]; Imo, 33,401 of 320,921 [10%]; Lagos, 37,217 of 320,921 [12%]; Nasarawa, 10,207 of 320,921 [3%]; and Rivers, 138,672 of 320,921 [43%]). State-based consortiums with government and nongovernmental organizations were established to improve local engagement, along with high-level engagement by U.S. Mission Nigeria (U.S. Embassy and Consulate in Nigeria) leadership.^¶^ Implementing partners reported weekly site-level data, beginning April 28, 2019. Data were distributed broadly to critical stakeholders (e.g., CDC Nigeria country office, U.S. CDC headquarters, and implementing partners) through an Excel-based dashboard, which was used to analyze data and adapt operations. Interstate learning was facilitated through weekly videoconferences among stakeholders and site visits.

Weekly ART Surge programmatic data and quarterly PEPFAR Monitoring and Evaluation Reporting data,** was analyzed to assess ART Surge progress across four areas: 1) case findings^††^ measured by the weekly number of positive test results and positivity rate (i.e., proportion of tests that were positive); 2) ART initiation, measured by the weekly number of newly identified persons with HIV infection who initiated ART and the rates of linkage to ART, using a proxy indicator calculated as the number of newly identified persons with HIV infection who initiated ART, divided by the number of positive test results; 3) annualized patient retention, measured using a proxy indicator calculated as the number of persons with HIV infection receiving ART as of March 31, 2020, divided by the sum of those receiving ART as of March 31, 2019, and the number of newly identified persons with HIV infection who initiated ART during April 1, 2019–March 31 2020; and 4) ART program growth, defined as the increase in total number of persons with HIV infection receiving ART between two time points. Persons with HIV infection receiving ART were defined as clients at a PEPFAR-supported site in one of the nine states with a maximum of 27 days since their last appointment; clients whose last appointment was ≥28 days earlier were considered to not be receiving ART. Medians were assessed overall and by state for each of the four programmatic areas, and positivity rates were assessed compared with NAIIS-estimated state prevalence. This activity was reviewed by CDC and was conducted consistent with applicable federal law and CDC policy.^§§^

During May 4, 2019–March 21, 2020, the weekly number of HIV tests conducted in the nine states that participated in the ART Surge program increased 500%, from 14,244 to 85,326 tests conducted, and the weekly number of positive test results increased 370%, from 622 to 2,929 persons with HIV infection identified (Figure 1). The overall median weekly positivity rate was 4.3% (range = 3.4% [March 21, 2020] to 5.4% [July 27, 2019]). The state median weekly positivity rate was above the estimated state prevalence (median positivity rate range = 2.5% in Nasarawa [estimated state prevalence = 1.6%] to 9.2% in Benue [estimated state prevalence = 4.3%]). The weekly number of newly identified persons with HIV infection who initiated ART increased 410% from 587 to 2,996 (Figure 2). The overall median weekly proxy for ART initiation was 101% (range = 93% [May 11, 2019] to 107% [September 28, 2019]). State median weekly proxies for ART initiation ranged from 96% in Lagos to 117% in Benue.^¶¶^ In the first 12 months of the ART Surge program, the number of persons with HIV infection receiving ART in the nine ART Surge states increased by 97,387 (30%), from 322,247 on March 31, 2019, to 419,634 on March 31, 2020 (Table). The annualized (March 2019–March 2020) proxy retention indicator was 99% overall, and the state proxy retention indicator ranged from 88% in Lagos to 117% in Gombe.***

**FIGURE 1:**
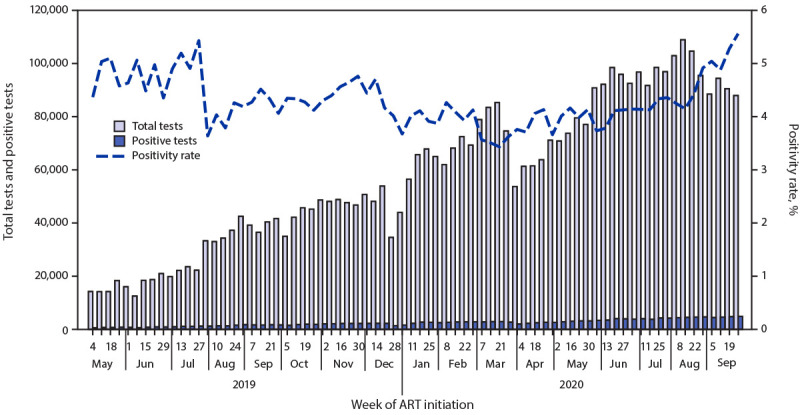
Total number of HIV tests conducted, positive test results, and positivity rate (%),* by week — nine Nigerian states^†^^,^^§^ participating in the Antiretroviral Therapy (ART) Surge program, May 4, 2019–September 26, 2020 * Positivity rate was calculated as the number of positive tests divided by the total number of tests conducted using weekly reported programmatic data. Reporting began the week ending with May 4, 2019. Reporting weeks end on Saturdays. ^†^ Benue, Delta, Enugu, Federal Capital Territory, Gombe, Imo, Lagos, Nasarawa, and Rivers. ^§^ On February 27, 2020, the Nigeria Centre for Disease Control confirmed the first confirmed COVID-19 case and activated an Emergency Operations Center on February 28. Thereafter, the government of Nigeria implemented COVID-19 mitigation efforts, including school closures (beginning March 19), international travel bans (beginning March 23), and statewide stay-at-home orders (beginning March 30).

**FIGURE 2:**
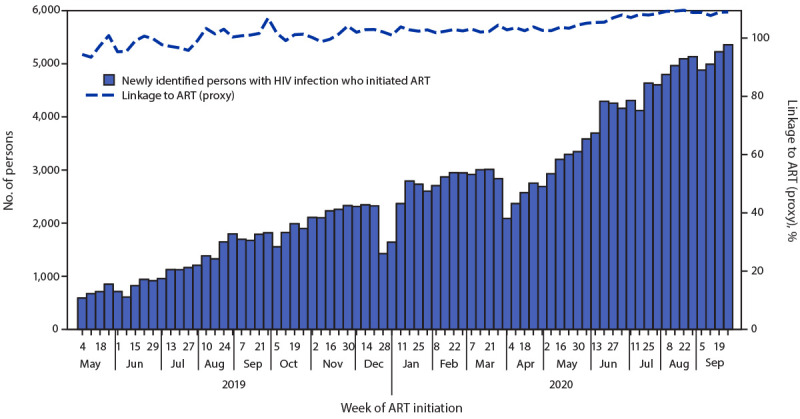
Number of newly identified persons with HIV infection who initiated antiretroviral therapy (ART) and proxy indicator for linkage to ART (%),* by week — nine Nigerian states^†^^,^^§^ participating in the ART Surge program, May 4, 2019–September 26, 2020 * Using weekly reported program data, a proxy indicator for ART initiation was calculated as the number of newly identified persons with HIV infection who initiated ART divided by the number of positive test results. ART initiation rates using the proxy indicator could exceed 100%, including if persons with HIV infection identified in one week did not initiate ART until the following week. Weekly programmatic data reporting began the week ending with May 4, 2019. Reporting weeks end on Saturdays. ^†^ Benue, Delta, Enugu, Federal Capital Territory, Gombe, Imo, Lagos, Nasarawa, and Rivers. ^§^ On February 27, 2020, the Nigeria Centre for Disease Control confirmed the first confirmed COVID-19 case, and activated an Emergency Operations Center on February 28. Thereafter, the government of Nigeria implemented COVID-19 mitigation efforts, including school closures (beginning March 19), international travel bans (beginning March 23), and statewide stay-at-home orders (beginning March 30).

**TABLE:** Number of persons with HIV infection receiving antiretroviral therapy (ART), total number of newly identified persons with HIV infection who initiated ART, and annualized proxy retention indicator, before and during the COVID-19 pandemic — nine Nigerian states participating in the Antiretroviral Therapy (ART) Surge program,* March 31, 2019–September 30, 2020

ART Surge program states	Before COVID-19 (April 2019–March 2020)					During COVID-19 (April 2020–September 2020)				ART Surge total (April 2019–September 2020)	
	No. of persons with HIV receiving ART		Newly identified persons with HIV infection who initiated ART^†^(total no.)	Program growth^§^ (% increase)	Proxy indicator for retention (%)^¶^	No. of persons with HIV infection receiving ART		Newly identified persons with HIV infection who initiated ART** (total no.)	Program growth^††^ (% increase)	Newly identified persons with HIV infection who initiated ART^§§^ (total no.)	Program growth^¶¶^(% increase)
	As of Mar 31, 2019	As of Mar 31, 2020				As of Jun 30, 2020	As of Sep 30, 2020				
**Total**	**322,247**	**419,634**	**102,497**	**97,387 (30)**	**99**	**461,574**	**530,449**	**109,398**	**110,815 (26)**	**211,895**	**208,202 (65)**
Benue	136,606	156,579	21,280	19,973 (15)	99	160,626	171,434	15,945	14,855 (9)	37,225	34,828 (25)
Delta	15,208	20,673	6,080	5,465 (36)	97	26,234	32,779	12,790	12,106 (59)	18,870	17,571 (116)
Enugu	18,110	22,893	5,640	4,783 (26)	96	25,035	28,783	6,307	5,890 (26)	11,947	10,673 (59)
Federal Capital Territory	38,185	44,901	8,161	6,716 (18)	97	48,777	57,007	11,520	12,106 (27)	19,681	18,822 (49)
Gombe	14,377	21,284	3,805	6,907 (48)	117	22,263	24,675	3,218	3,391 (16)	7,023	10,298 (72)
Imo	12,057	16,945	5,174	4,888 (41)	98	19,104	21,162	4,650	4,217 (25)	9,824	9,105 (76)
Lagos	25,291	31,538	10,535	6,247 (25)	88	38,404	44,819	12,448	13,281 (42)	22,983	19,528 (77)
Nasarawa	36,372	42,780	7,419	6,408 (18)	98	44,865	50,057	6,439	7,277 (17)	13,858	13,685 (38)
Rivers	26,041	62,041	34,403	36,000 (138)	103	76,266	99,733	36,081	37,692 (61)	70,484	73,692 (283)

* The ART Surge program is supported by CDC through the U.S. President’s Emergency Plan for AIDS Relief (PEPFAR).

^†^ Includes all newly identified persons with HIV infection who initiated ART during April 2019–March 2020.

^§^ Difference between the number of persons with HIV infection receiving ART on March 31, 2019, and March 31, 2020.

^¶^ PEPFAR Monitoring and Evaluation Reporting quarterly data; the annualized proxy retention indicator is calculated as the number of persons with HIV infection receiving PEPFAR-supported ART as of March 31, 2020, divided by the sum of the number of persons with HIV infection receiving PEPFAR-supported ART as of March 31, 2019, and the number of newly identified persons with HIV infection who initiated ART during April 1, 2019–March 31, 2020. The proxy indicator does not directly measure the number of persons with HIV infection retained on ART, and persons with HIV infection entered into the ART program for reasons other than ART initiation (e.g., re-engaged persons with HIV infection). Thus, >100% retention is possible.

** Includes all newly identified persons with HIV infection who initiated ART during April 2020–September 2020.

^††^ Difference between the number of persons with HIV infection receiving ART on March 31, 2020, and September 30, 2020.

^§§^ Includes all newly identified persons with HIV infection who initiated ART during April 2019–September 2020.

^¶¶^ Difference between the number of persons with HIV infection receiving ART on March 31, 2019, and September 30, 2020.

ART Surge activities were affected by the COVID-19 pandemic during March–May 2020,^†††^ with fewer tests conducted, fewer positive test results, and fewer newly identified persons with HIV infection who initiated ART. Following Nigeria Centre for Disease Control guidelines, the ART Surge program implemented COVID-19 mitigation measures, including provision of face masks for staff members, enhanced hand hygiene by staff members and clients during clinical visits, and physical distancing measures (e.g., staggered clinical appointments, 2-meter spacing between seating). Given limitations in facility-based services, community-based activities were increased through mobile teams, toward strengthened case finding, ART initiation, and patient retention. By May 16, 2020, ART Surge activities returned to prepandemic levels (i.e., those before March 21, 2020) and continued to increase (Figure 1) (Figure 2). During April 2020–September 2020, 109,398 persons with newly identified HIV infection initiated ART and the number of persons with HIV infection receiving ART increased by 110,815 (26%) (from 419,634 [March 31, 2020] to 530,449 [September 30, 2020]) (Table).

## Discussion

During May 2019–September 2020, ART Surge activities in nine Nigerian states resulted in an approximate eightfold increase in the weekly number of newly identified persons with HIV infection who initiated ART and a 65% increase in the total number of persons (208,202) with HIV infection receiving PEPFAR-supported ART. During April–September 2020 alone, ART Surge activities resulted in an increase of 26% in the total number of persons (110,815 ) with HIV infection receiving ART across nine Nigerian states, demonstrating rapid program adaptation during the COVID-19 pandemic. These increases accelerated progress toward achieving the Joint United Nations Programme on HIV/AIDS (UNAIDS) targets (*5*). Estimates from the NAIIS were crucial in identifying states and substate geographic units with a large estimated number of persons with HIV infection who were not receiving ART (*1*). In addition, the ART Surge program’s incident command structures provided flexible management of operations, and weekly data disseminated through a user-friendly dashboard allowed for data-guided, locally adapted interventions and improved accountability. Collaboration among ART Surge states facilitated interstate learning and helped disseminate successful interventions to achieve broader implementation. Finally, diplomatic engagement with governors and local leaders helped combat HIV stigma, supported the elimination of user fees (e.g., registration fees and folder fees), and assisted with the provision of rapid test kits from local stakeholders.

Despite this progress, many persons with HIV infection in Nigeria remain unaware of their status and are not receiving ART (*1*). Additional interventions could help improve case finding, ART initiation, and patient retention. For example, in October 2020, through PEPFAR, CDC expanded the ART Surge program strategies to nine additional states.^§§§^ Data analysis that supports programmatic activities, such as case finding and rapid linkage to treatment, might help identify more persons with HIV infection who are not receiving ART, including female sex workers and men who have sex with men, who are among the populations at higher risk for infection. As more persons with HIV infection receive ART, adherence, patient retention, and viral load suppression remain critical. In addition, preventive measures to minimize losses and reengage persons with HIV infection who miss appointments, or have dropped out of care, are important to reach and maintain ART coverage targets. Site-level improvements adapted to local needs and preferences are also important.

The findings in this report are subject to at least four limitations. First, despite continual PEPFAR data quality assurance activities, data quality might be affected by reporting challenges (e.g., site-level electricity or Internet outages preventing data transmission). Second, newly identified patients with HIV infection were categorized by client self-reporting; therefore, the actual proportion of newly identified persons with HIV infection who initiated ART is unknown. Third, proxy indicators derived from aggregate program data were used to evaluate ART initiation and patient retention, which might vary from patient-level analysis. Finally, persons with HIV infection can access health services at any site, regardless of state of residence; therefore, some persons might have been counted more than once, which limited direct assessment of ART coverage.

Despite the challenges of the COVID-19 pandemic, CDC’s ART Surge program has accelerated progress toward HIV epidemic control in Nigeria, which is aligned with UNAIDS targets (*5*). Comprehensive, data-guided, locally adapted interventions and incident command structures can help increase and retain the number of persons with HIV infection who receive ART, reducing HIV-related morbidity and mortality as well as decreasing HIV transmission.

**Summary**

**What is already known about this topic?**

In 2018, an estimated 1.8 million persons living in Nigeria had HIV infection. Through the U.S. President’s Emergency Plan for AIDS Relief (PEPFAR), CDC launched an 18-month antiretroviral therapy (ART) Surge program in nine Nigerian states in April 2019, including implementation of incident command structures to manage operations.

**What is added by this report?**

The weekly number of persons with newly identified HIV infection who initiated ART increased approximately eightfold, from May 4, 2019, to September 26, 2020. Compared with March 2019, a total of 208,202 more persons were receiving PEPFAR-supported ART in September 2020.

**What are the implications for public health practice?**

Comprehensive, data-guided, locally adapted interventions and use of incident command structures can increase the number of persons with HIV infection who receive ART, reducing mortality and decreasing HIV transmission.

## Footnotes

*
https://www.state.gov/pepfar/

† Among the NAIIS-derived estimated 1.8 million persons with HIV infection in Nigeria, 1.1 million were receiving ART; according to PEPFAR program data, 709,654 persons with HIV infection were receiving PEPFAR-supported ART as of December 31, 2018.

§ The Institute of Human Virology, Nigeria; the Centre for Integrated Health Program, Abuja, Nigeria; the Catholic Caritas Foundation Nigeria, and the AIDS Prevention Initiative in Nigeria.

¶ Since August 2019, senior leaders from the U.S. Mission Nigeria (including the ambassador, deputy chief of mission, and consul general), and country directors for the U.S. CDC and the U.S. Agency for International Development, engaged state governors, religious and traditional leaders, and civil society groups. Engagement activities included outreach to destigmatize HIV, advocacy to eliminate user fees for persons seeking HIV treatment, and requests for assistance with the provision of rapid test kits. Diplomatic engagement enlisted state governors in Benue, Imo, Lagos, Nasarawa, and Rivers to support ART Surge activities, resulting in increased health resources for and knowledge of the CDC ART Surge program.

** Weekly programmatic data reporting began the week ending on May 4, 2019. Reporting weeks end on Saturdays. All PEPFAR-supported countries are required to report Monitoring and Evaluation Reporting data quarterly.

†† Case finding interventions included prioritization of substate geographic units with the largest estimated ART coverage gap; enhanced use of a nationally validated risk stratification tool to screen persons with higher risk for HIV infection; engagement of core community-based organizations; targeted community testing; and programming based on weekly reported data.

§§ 45 C.F.R. part 46.102(l)(2), 21 C.F.R. part 56; 42 U.S.C. Sect. 241(d); 5 U.S.C. Sect. 552a; 44 U.S.C. Sect. 3501 et seq.

¶¶ A proxy indicator for ART initiation was calculated as the number of newly identified persons with HIV infection who initiated ART divided by the number of positive test results. As such, ART initiation rates using the proxy indicator could exceed 100% if persons with HIV infection identified in 1 week did not initiate ART until the following week.

*** The proxy indicator did not directly measure the number of persons with HIV infection retained on ART, and the total number of persons with HIV infection receiving PEPFAR-supported ART included persons with HIV who entered into the ART program for reasons other than ART initiation (e.g., reengaged persons with HIV infection). Thus, >100% retention was possible.

††† On February 27, 2020, the Nigeria Centre for Disease Control confirmed the first confirmed COVID-19 case and activated an Emergency Operations Center on February 28. Thereafter, the Government of Nigeria implemented COVID-19 mitigation efforts, including school closures (beginning March 19), international travel bans (beginning March 23), and statewide stay-at-home orders (beginning March 30).

§§§ In addition to the nine ART Surge states (Benue, Delta, Enugu, Federal Capital Territory, Gombe, Imo, Lagos, Nasarawa, and Rivers), in October 2020, through PEPFAR, CDC expanded the ART Surge program strategy to the following nine states: Ekiti, Kaduna, Katsina, Kogi, Ogun, Ondo, Osun, Oyo, and Plateau.
